# Reviewed article: Souza IG, Duarte YAO, Nascimento MMG, Molino CGRC, Rezende CP, Santos JLF. Competições terapêuticas e fragilidade em pessoas idosas no município de São Paulo: estudo transversal de base populacional, 2015. Epidemiol Serv Saude. 2025:34;20240104. 10.1590/S2237-96222025v34e20240104.en

**DOI:** 10.1590/S2237-96222025v34e20240104.b

**Published:** 2025-04-11

**Authors:** Clineu Mello Almada

**Affiliations:** 1 Universidade Federal de São Paulo Hospital São Paulo, Medicina, São Paulo, SP, Brazil

## Peer review B

## Questionnaire

Does the manuscript contain new and significant information to justify publication? Yes

Does the Abstract (Summary) clearly and accurately describe the content of the article? Yes

Is the problem significant and concisely stated? Yes

Are the methods described comprehensively? Yes

Are the interpretations and conclusions justified by the results? Yes

Is adequate reference made to other work in the field? Yes

## Manuscript Structure

Length of article is: Adequate

Number of tables is: Adequate

Number of figures is: Adequate

**Please state any conflict(s) of interest that you have in relation to the review of this paper (state “none” if this is not applicable).**

None

**Interest:** Excellent

**Quality**: Good

**Originality:** Excellent

**Overall:** Good

**Recommendation:** Accept

## Comments

A apresentação do trabalho está muito boa, utilizando uma linguagem clara e direta. A metodologia foi muito bem apresentada, inclusive ressaltando o cuidado metodológico no estudo longitudinal, do qual os dados foram analisados. Os resultados expressam dados de nossa realidade e a discussão destes é muito apropriada. Esse trabalho traz importante contribuição, na forma de alerta à prática clínica e pode influenciar diretrizes e medidas para políticas públicas de saúde. Parabéns aos autores!

